# A Novel Procedure of Total Organic Carbon Analysis for Water Samples Containing Suspended Solids with Alkaline Extraction and Homogeneity Evaluation by Turbidity

**DOI:** 10.3390/ijerph17113901

**Published:** 2020-05-31

**Authors:** Han-Saem Lee, Jin Hur, Yu-Hoon Hwang, Hyun-Sang Shin

**Affiliations:** 1 Department of Environment Energy Engineering, Seoul National University of Science and Technology, Seoul 01811, Korea; hansun213@seoultech.ac.kr (H.-S.L.); yhhwang@seoultech.ac.kr (Y.-H.H.); 2 Department of Environment and Energy, Sejong University, Seoul 05006, Korea; jinhur@sejong.edu

**Keywords:** total organic carbon, suspended solids, ultrasonication, alkaline extraction, turbidity, analytical procedure

## Abstract

This study was conducted to develop and validate a more reliable total organic carbon (TOC) analytical procedure for water samples containing suspended solids (SS). The effects of the combined ultrasonic and alkaline pretreatment (CULA) on the TOC measurement were studied in water samples containing SS from three origins (algae, sewage particles, and soil) under different analytical conditions (SS concentration, oxidation methods, and sieve size). The applicability of turbidity as a homogeneity index was also evaluated. With CULA, TOC recovery remained high (>80%) for SS concentration ranges up to four times larger than ultrasonic pretreatment alone (UL) due to enhanced particulate organic carbon (POC) solubilization, and did not significantly differ depending on the oxidation methods, at low SS concentrations, or with varying sieve sizes. In particular, the turbidity change rate (i.e., NTU_5_/NTU_0_) of the pretreated water sample showed a high correlation with TOC precision (*r*^2^ = 0.73, *p* < 0.01), which suggests that turbidity can be used as an indicator of sample homogeneity. A novel TOC analytical procedure is expected to be useful for more accurate assessments of the impact of particulate pollutants on water quality than current methods, and for the analysis of the carbon cycle, including POCs, in the environment.

## 1. Introduction

Total organic carbon (TOC) concentration in samples is widely used as an indicator of organic carbon behavior when evaluating water pollution, soil and sediment organic matter, and the carbon cycle [1,2,3,4]. As factors that affect water quality, such as natural processes (e.g., precipitation and weathering) and artificial activities (e.g., agricultural, urban, and industrial), gradually diversify and water consumption increases, reliable water quality indicators for water management are becoming more important [5,6,7,8,9]. In particular, TOC has attracted attention as an indicator of non-biodegradable organic matter including particulate pollutants from various non-point sources; it is being considered in lieu of the chemical oxygen demand (COD) test [10,11,12,13]. Many studies have also used TOC to understand the carbon cycle system, which is a key part of climate change caused by global warming [3,14,15].

TOC includes both dissolved organic carbon (DOC) and particulate organic carbon (POC), which have a difference in particle size of 0.45 µm [5,13]. Unlike DOC measurements, the reliability of TOC measurements has been continuously problematic because of a lack of homogenization in samples with high POC content. Aiken et al. [16] demonstrated that the TOC recovery in samples containing suspended solids (SS) ranged from 50–60% through an analysis of various commercial TOC instruments and wet oxidation (e.g., UV-persulfate (UVP) has a 10–20% lower TOC recovery than high-temperature combustion (HTC)). Kim et al. [17] noted that a higher SS concentration reduces the homogeneity of a water sample, which is a major factor in the reduced reliability of the TOC test because of the effects on sample dilution and sieve size.

Ultrasonication (UL) is conventionally used to improve the low homogeneity of water samples containing SS and TOC recovery [18,19,20]. However, although homogenization may vary depending on the sample origins, SS concentration, and analytical conditions (e.g., sieve size, oxidation method, etc.), there are no detailed guidelines for analytical conditions and procedures for water samples containing SS. For example, in South Korea, the standard analysis method recommends homogenizing samples by sieving to a 300-µm particle size after pretreatment with 20–40 kHz of ultrasonication [18]. The EPA 415.2 method [21] of the United States Environmental Protection Agency (EPA) sets a particle size of <200 µm for TOC analysis with UVP oxidation. In contrast, the EPA 415.3 method [22] does not set a specific sieve size and suggests that floating vegetation, animal matter, volatile organic matter, and settleable solids should be excluded from the TOC value. The International Organization for Standardization (ISO) does not restrict the particle size for TOC analysis and advises measuring only DOC if the TOC measurements have low reproducibility (i.e., relative standard deviation (RSD) > 10%). Therefore, for samples containing SS, the development of sample pretreatment and evaluation methods to achieve sample homogeneity while minimizing the influence of various experimental factors is important for the improvement of the reliability of TOC measurements.

Alkaline extraction is widely used for extracting organic matter from particles in various research fields, such as carbon cycle studies and organic waste recycling [15,23,24,25,26]. For example, He et al. [27] reported that the distribution and behavior of dissolved organic matter (DOM) and particulate organic matter (POM) in sediment can be more accurately assessed by comparing and evaluating DOM in pore water and alkaline-extracted DOM from sediment. Lu et al. [28] noted that when alkaline extraction was combined with ultrasonic treatment of sewage sludge, the biogas yield was improved because of increased anaerobic digestion efficiency as a result of the reduced molecular weight of the POM in the sludge. Although alkaline extraction is known to be effective for extracting and separating POM and for the pretreatment of particulate samples, the application of alkaline extraction in sample pretreatment methods for TOC analysis has received little attention.

Recently, we established proper alkaline pretreatment conditions by evaluating the effect of alkaline extraction for TOC measurement on water samples containing SS from different origins and reported that the simultaneous treatment of alkalis and ultrasonics (i.e., combined alkaline and ultrasonic pretreatment method, CULA) could significantly improve the accuracy and precision of TOC measurements better than conventional ultrasonic pretreatment (UL) [29]: the TOC recovery improved approximately two-fold with CULA (87.6%) compared with UL (34.7%) [29]. However, as mentioned above, for the new pretreatment method to be applied to the actual application stage with various sample matrices and/or analytical conditions, studies on further verification are required for the effectiveness of CULA on the analytical conditions (such as SS concentration, sieve size, and the oxidation method) and a detailed TOC analysis procedure including the evaluation of pretreatment efficiency is also necessary. Additionally, there is no method for evaluating sample homogeneity in the pretreatment step. Therefore, to address the growing demand for real-time online measurements, a method must be developed to evaluate the homogeneity of water samples containing SS with greater speed and efficiency. At present, the only method proposed for determining sample homogeneity is in DIN EN 1484 [19], which compares two TOC values collected from the top and bottom of a beaker containing the sample. This method has the disadvantage of taking a long time because sample homogeneity is determined through post-analysis.

In this study, the effects of CULA on varying TOC analytical conditions that should be set or regarded in the execution state, such as the oxidation methods, SS concentration, and sieve size, were analyzed for water samples containing SS with different origins (algae, sewage particles (SP), and soil), and the results were compared and evaluated with the results of UL. Also, to evaluate the feasibility of using turbidity as a sample homogeneity index, the precision (RSD, %) and standard error (SE, %) of the TOC recovery and changes in the turbidity according to the pretreatment method were investigated. The main purpose of this study was to present a novel TOC analysis procedure that is more reliable than current methods for SS-containing samples, including sample pretreatment and homogeneity evaluation.

## 2. Materials and Methods 

### 2.1. Water Samples Containing SS

The origins of the SS in this study were algae, SP, and soil. The algae were collected and concentrated using a plankton net with a 20-µm pore size at Uiam Dam (37°50′16.8″ N, 127°40′33.5″ E) before the rainy season in May and early July 2017. The SE was collected from the influent of a public wastewater treatment plant in South Korea (37°43′03.9″ N, 127°03′04.9″ E). The topsoil (0–10 cm depth) sample was also collected near Uiam Dam. All collected particle samples were freeze-dried after foreign substances (e.g., roots, twigs, and plastic pieces) were removed. Finally, the particle samples were sieved with a 2.0 mm mesh. The carbon contents of the pretreated particle samples were determined by elemental analysis (vario MACRO Cube, Elementar Ltd., Germany) to be 45.08% for algae, 41.94% for SP, and 1.62% for soil. The water samples containing SS with different origins were prepared by dispersing the pretreated particle samples in distilled water.

### 2.2. Pretreatment Experiments

The SS-containing water samples were separated into equal portions from a stock solution that was continuously stirred to minimize the differences among the samples. The SS concentrations of the solution were set to 50 mg/L for algae, 30 mg/L for SP, and 1000 mg/L for soil, respectively, to be in a similar TOC concentration range (10–20 mg-C/L). The SS-containing solution was pretreated using the ultrasonic homogenization method of Kim et al. [30]. Briefly, 200 mL of the solution was irradiated with ultrasound for 10 min and then sieved to 200-µm particle size to prevent tube blockage in the TOC instrument. A probe-type ultrasonic device (Ultrasonic Processor JY92-IIN, Scientz, China) with a titanium tip of ϕ6 mm was used. The output power of the device was set to a maximum of 650 W with a pulse ratio of 20 (i.e., on-time/off time: 100 s/5 s). The frequency was 20 kHz. Alkaline extraction was then combined with UL as follows. A NaOH solution was mixed with the SS-containing solution at a ratio of 1:10 to obtain 0.01 mol/L NaOH for UL under the conditions presented above [28,29]. The NaOH concentration (0.01 mol/L) was confirmed in our previous research as a suitable condition in which a small amount of salt scale can be generated in an oxidizing device while maintaining a high TOC recovery rate [29]. The pretreated solution was immediately neutralized with an HCl solution to prevent further extraction. To evaluate the effect of the SS concentration, the SS stock solution was diluted 1–20 times depending on the origin. Sieving was further carried out at 100, 200, 300, and 500 µm to investigate the effect of the particle size on TOC recovery. The homogeneity of the sample after pretreatment was evaluated according to the DIN EN 1484 method [19]. Pretreated samples were taken from the upper part (2/10 position down from the water surface) and the lower part (2/10 position from the bottom) of the sample container (300 mL glass beakers, DIAMOND Co.). All experiments were conducted in triplicate.

### 2.3. TOC, Particle Distribution, and Turbidity Analyses

For the TOC analysis, the vario TOC select (Elementar, Germany) was used with HTC oxidation and the DE/multi N/C (Jena, Germany) with UVP. The TOC was measured with the non-purgeable organic carbon method in particle mode with continuous stirring to prevent particle settlement [13,31]. The particle sizes in the SS-containing solution were analyzed with a laser diffraction particle size analyzer (Mastersizer 3000, Malvern Co., UK). The turbidity of the solution was measured with a portable turbidimeter (2100P, HACH Co., USA). The turbidity change based on particle precipitation was measured without the solution being shaken at a constant settlement time (1, 3, 5, 10, 20, 30 min) after pretreatment regardless of the sample type.

### 2.4. Statistical Analysis

Statistical analyses were performed using the SPSS statistics program (Version 18.0 ) (IBM, Armonk, NY, USA). Paired t-tests were carried out to verify the differences in TOC recovery between the HTC and UVP methods (α < 0.05) and to evaluate the homogeneity of the pretreated sample. Two-way analysis of variance (ANOVA) and Tukey’s tests for post-hoc analysis was performed to evaluate the influence of the sieve size and oxidation method on the application of the alkaline extraction method (α < 0.05).

## 3. Results and Discussion

### 3.1. Validation of Combined Ultrasonic and Alkaline Pretreatment

To clarify the basis for the improvement of the TOC measurement efficiency by the CULA, it was compared to UL in terms of TOC recovery (%), RSD (%), DOC/POC ratio, and particle size (Table 1). The CULA resulted in higher TOC recovery (77.5–95.4%) and higher precision (1.5–7.9%) than UL. The improved TOC measurement using the alkaline extraction method was confirmed by the change in the DOC/POC ratio and particle size distribution of samples containing SS with different origins. The DOC/POC ratios were 3.5 and 12 times higher for the algae and SP origin samples, respectively, with CULA compared to UL. The particle size distribution <200 µm was <70% with UL, whereas it was >99.9% for SP and soil with CULA. The algae origin sample showed that the majority of the TOC recovery (95.4%) was due to the DOC component (i.e., DOC/POC = 11.50), so the effect of sieve-filtered particles >200 µm (i.e., 38.2%) on TOC measurements was almost imperceptible. From the above results, it was confirmed that the accuracy and precision improvements of the TOC measurements by CULA were due to the enhanced homogeneity of the sample and oxidation rate of the organic matter through the reduction of the SS particle size and by increasing the conversion rate of POC into DOC.

### 3.2. Effect of CULA on TOC Recovery with Different Oxidation Methods

**Figure 1:**
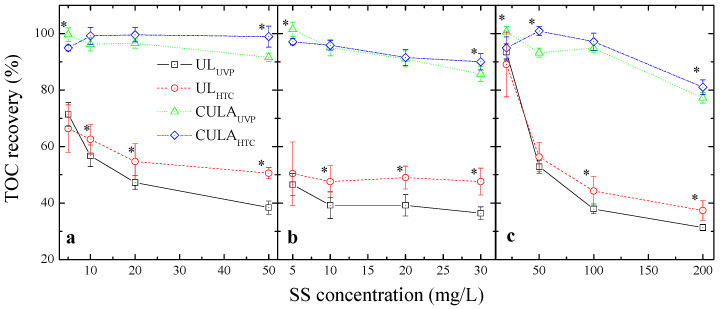
Changes in total organic carbon (TOC) recovery (%) of water samples containing particles from different origins compared between different pretreatment methods (UL: ultrasonication only, CULA: combined ultrasonic and alkali pretreatment), oxidation methods (UVP: UV-persulfate, HTC: high-temperature combustion), and suspended solids (SS) concentration: (**a**) Algae; (**b**) Sewage particles (SP); and (**c**) Soil. * indicates a statistical difference between UL and CULA pretreatments (*p* < 0.05, *n* = 18).

To evaluate the effect of CULA on TOC recovery with different oxidation methods, the change in TOC recovery was analyzed according to the SS concentration (Figure 1). The TOC recovery (%) was significantly lower in all samples that used UL compared to CULA and decreased further with increased SS concentrations. With HTC oxidation, TOC recovery of the algae and SP samples decreased by 11.7% and 2.8%, respectively, when the SS concentration was increased from 5 mg/L to 20 mg/L. For soil, the TOC recovery also greatly decreased (by 51.7%) when the SS concentration was increased from 20 mg/L to 200 mg/L. This reduction in TOC recovery with increasing SS concentration was higher with UVP than HTC (i.e., 24.2% for algae, 7.3% for SP, and 62.1% for soil). The difference in TOC recovery based on the oxidation method (i.e., HTC vs. UVP) exceeded 10% with SS concentrations of over 50 and 20 mg/L for algae and SP, respectively. In the case of soil, there was a significant difference when the SS concentration was >100 mg/L (approximately 6.0%) (*p* < 0.05, *n* = 18). These results suggest that a higher SS concentration reduces the sample pretreatment effect of UL and that this influence is greater with the UVP oxidation method than with HTC. In contrast, there was no significant difference in TOC recovery based on the oxidation method at low SS concentrations (i.e., algae and SP < 5 mg/L, soil < 50 mg/L). This indicates that if the SS concentration is sufficiently low, UVP can be applied as effectively as HTC when performing TOC analysis of SS-containing water samples. Similarly, Kim et al. [17] reported that the UVP method showed a better detection limit and precision for sewage influent and effluent samples with low SS content (i.e., < 6 mg/L) than the HTC method. They concluded that the higher precision of TOC values with UVP under low SS conditions was due to the higher sample loading compared to HTC.

In contrast, CULA showed a higher TOC recovery (>80%) than UL and greatly mitigated the reduced TOC recovery due to the increased SS concentration and differences in TOC recovery depending on the oxidation method. The TOC recovery at relatively low SS concentrations (algae and SP < 10 mg/L, soil < 100 mg/L) was approximately 95% for all samples. The reduction in TOC recovery with an increase in the SS concentration was greatly mitigated (10–20%). The limit of the SS concentration did not show a significant difference (*p* < 0.05, *n* = 18) in TOC recovery between the two oxidation methods, i.e., it was <20 mg/L for algae and SP and 100 mg/L for soil with CULA. The SS concentration range was four times larger with CULA than with UL (5 mg/L for algae and SP, and 50 mg/L for soil). Thus, CULA may be acceptable for TOC measurements. These results were confirmed through a statistical comparison of the TOC recovery (%) for all SS origin samples using both oxidation methods (Table 2). The TOC recovery with UL was 36.3 ± 7.2% and 45.4 ± 9.3% with UVP and HTC, respectively (*p* < 0.05, *n* = 72). It was similar with CULA at 86.4 ± 8.7% and 89.8 ± 8.7% with UVP and HTC, respectively (*p* = 0.17, *n* = 72). The TOC recovery with CULA satisfies the generally accepted quality control criterion (i.e., >80%, ISO 20236, 2018). This result was due to alkaline extraction, which increased the DOC to POC ratio and reduced the particle size. Therefore, with CULA, both the UVP and HTC oxidation methods can be used for a wider SS concentration range.

### 3.3. Effect of CULA on TOC Recovery with Different Sieve Sizes for Sample Homogenization

Two-way ANOVA was performed on the TOC recovery results with CULA according to the sieve size (100–500 µm) to quantify the effect of particle size variation on TOC recovery following sample pretreatment (Table 2). The upper particle size limit (500 µm) was selected based on the consideration of the inner diameter limit (700 µm) of the sample delivery tube of the TOC instrument used in this study. The results show that the sieve size had a significant effect on the TOC recovery with UL (*p* < 0.05, *n* = 72). The sieve size of 100 µm showed a TOC recovery of 35.3%, which was significantly different from the TOC recovery (42.4–44.8%) at a sieve size of 200–500 µm (*p* < 0.05, *n* = 72). This suggests that different TOC results can be obtained depending on the particle size after sample homogenization using UL. Therefore, the ultrasonic homogenization method requires a prior review of the TOC levels based on sieve size before SS-containing water samples can be analyzed. In contrast, the sieve size did not have a significant effect on TOC recovery with CULA (*p* = 0.57, *n* = 72). The TOC recovery was similar at different sieve sizes (100–500 µm) with a range of 86.4%–89.6% [18,20]. This suggests that CULA can increase the homogeneity of SS and eliminate the uncertainty regarding TOC levels according to the sieve size.

### 3.4. Homogeneity of Water Samples Containing SS Depending on Turbidity

The RSD (%) and SE (%) of the TOC recovery and changes in the turbidity according to the pretreatment method were investigated to evaluate the relationship between the homogeneity and turbidity of SS-containing samples (Table 3). For comparison, the homogeneity results of samples taken from the upper and lower layers of the pretreatment sample container, according to the DIN EN 1484 method [19], are presented here. With UL, the differences between the TOC recoveries at the upper and lower sampling points were 4.5–7.9%, depending on the SS origin, after 10 min. After 30 min, the differences decreased to 0.42–2.16%. With CULA, the difference in TOC recovery between the sampling points was 0.90–2.18% after only 10 min. These results suggest that CULA can satisfy the previous homogeneity assessment standard [19] with a shorter pretreatment time than UL. This was also confirmed through a comparison of RSD (%) and SE (%) of the repeated samples. With UL, the precision of the TOC recovery after 10 min was 15.7–39.9%. With CULA, the precision was 4.4–9.5% after 10 min. This indicates that CULA meets the quality control requirements (RSD < 10%) of international standards for TOC analysis [20]. The SE was also 2.5–12.2% with CULA, which was a significant improvement compared to the SE of 8.8–17.9% with UL.

**Figure 2:**
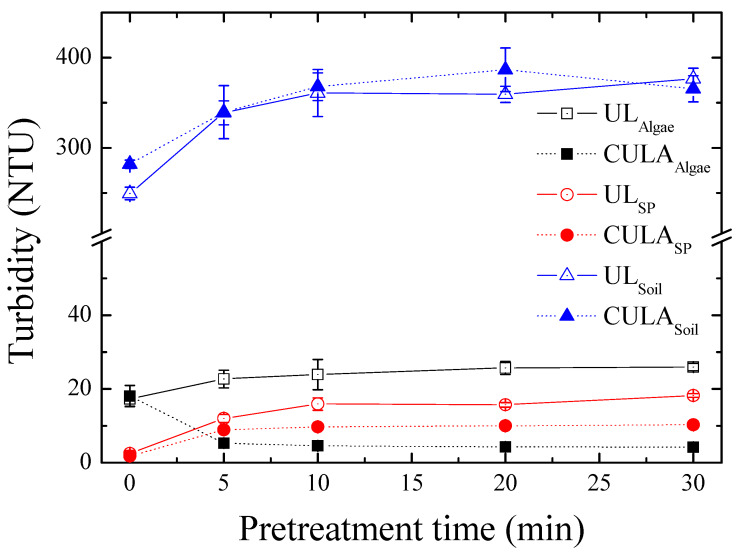
Changes in turbidity (NTU) of water samples containing suspended solids from different origins (algae, sewage particles (SP), and soil) for pretreatment time (min).

The DIN EN 1484 evaluation method [19] is cumbersome because sampling for analysis and measurement takes a relatively long time. Specifically, it has limited applicability for real-time analysis of field samples, such as in an online automatic measurement system. Therefore, a more effective method for evaluating the homogeneity of pretreated SS-containing samples is needed. This study investigated the correlation between the change in turbidity and precision according to the time of pretreatment (UL and CULA) for water samples containing SS with different origins to determine the potential of turbidity as an indicator of sample homogeneity (Figure 2). Turbidity fluctuated for the treatment time depending on the sample origin and pretreatment method, however, it became constant for all origins after 10 min. This means that the suspended particles affecting turbidity were homogenized after 10 min of treatment, which is consistent with previously reported results for the change in TOC recovery of SS-containing samples for the UL time [30]. The decrease in turbidity of algae (organic content (OC), 45.08%) with CULA was attributed to the reduction of particulates affecting turbidity from the dissolution of POM into dissolved organics (i.e., DOC/POC = 11.50; see Table 1). The SP with high OC (41.9%) also increased by 3.5 times in the POC to DOC conversion rate with CULA compared to UL, which decreased the sample turbidity.

**Figure 3:**
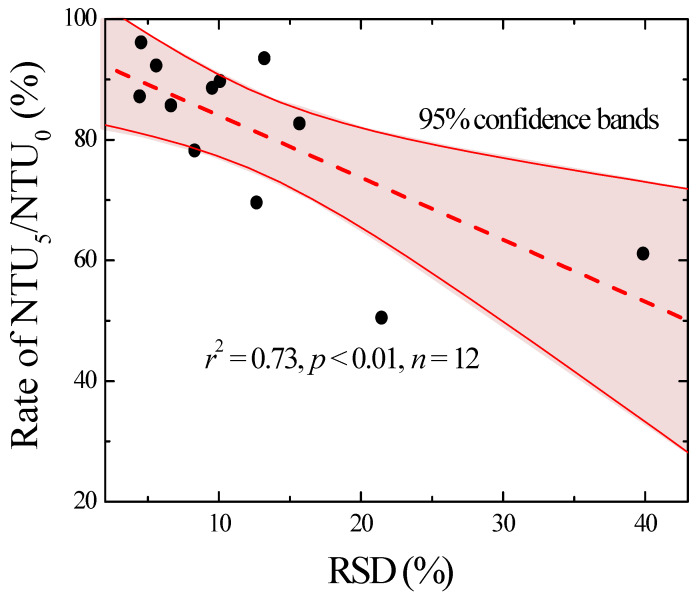
Correlation between the relative standard deviation (RSD, %) and the percent ratio of NTU_5_ (turbidity of the sample after 5 min) to NTU_0_ (initial).

Based on the understanding that the turbidity itself depends on the characteristics of the sample origins and sample pretreatment method (Table 3), the effect of turbidity on the sample homogeneity was evaluated according to the turbidity change rate (i.e., NTU_x_/NTU_0_). This corresponds to the ratio of the turbidity (NTU_1_, NTU_3_, NTU_5_) measured after a certain settlement time (i.e., 1, 3, or 5 min) of the pretreated sample to the initial turbidity (NTU_0_). The turbidity change rate at the settlement time of 1 min did not show significant differences between the two pretreatment methods and was in the range of 81.3–99.2% depending on the sample origin. At settlement times of 3–5 min, the mean and coefficient of variation (CV, %) of the turbidity change rate were 88.7% and 6.3% respectively, with CULA, and 78.7% and 18.5%, with UL. These results demonstrate that CULA maintained more homogenous samples than UL by reducing the turbidity change rate and the non-uniformity of the treated sample due to SS sedimentation. The turbidity change rate and RSD showed low correlations at settlement times of 1 min (*r*^2^ = 0.15, *p* < 0.64) and 3 min (*r*^2^ = 0.51, *p* < 0.09), but showed a significant correlation with a settlement time of 5 min (*r*^2^ = 0.73, *p* < 0.01) (Figure 3). These results suggest that the turbidity change rate at 5 min (i.e., NTU_5_/NTU_0_) can be used to evaluate the homogeneity of SS-containing water samples, and the homogenization process should be carried out until the turbidity change rate is >85% to meet the general precision requirement (RSD < 10%). The above results confirmed that sample homogeneity, which directly affects the precision of the TOC measurement of SS-containing samples, can be easily assessed using the turbidity change rate regardless of the sample origin and pretreatment method.

### 3.5. Proposed TOC Analytical Procedure Using Alkaline Extraction and Turbidity Assessment

**Figure 4:**
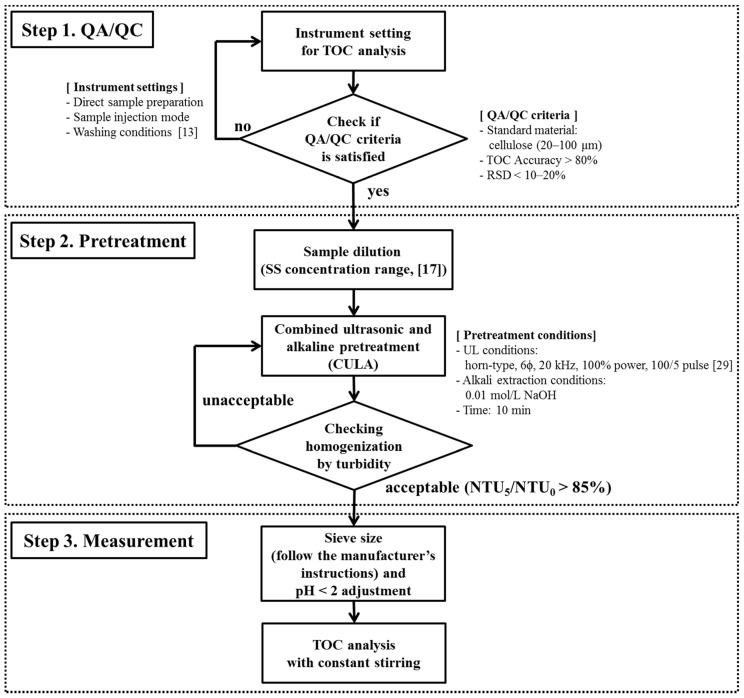
Proposed procedure for total organic carbon (TOC) analysis of water samples containing suspended solids (SS). RSD—relative standard deviation.

A new TOC analytical procedure is proposed for improving the homogeneity of water samples containing SS by using CULA and a turbidity assessment (Figure 4). To cover the entire process, some of the instrument setting-procedures for quality assessment and quality control using SS standard samples (cellulose, 20–100 µm) published by this laboratory are also included [13]. First, the cellulose standard solution is used to set up the equipment cleaning conditions and sample injection modes that meet international quality control standards (i.e., accuracy > 80%, precision < 10%) for the TOC analysis of water samples containing SS. Second, after the sample is diluted to the appropriate concentration according to the SS origin, it is pretreated with CULA. The adequacy of the sample homogeneity is then determined by the change in the turbidity rate of the sample after pretreatment (i.e., NTU_5_/NTU_0_ > 85%). Third, the homogenized sample is placed in the oxidation chamber for TOC measurement after the removal of impurities (sieving according to the device manufacturer’s instructions) that may block the delivery tube. The HTC method is preferred for oxidation, whereas the UVP method is also applicable for samples with low SS concentrations (e.g., algae and SP < 20 mg/L, soil < 200 mg/L). The proposed analytical procedure with the CULA pretreatment can effectively address the major limitations of conventional TOC analysis such as ensuring sample homogeneity, obtaining a high TOC measurement reliability (TOC recovery and precision), and minimizing the influence of various experimental factors (SS concentration, oxidation method, and sieve size). Turbidity, in particular, as an indicator can be applied to ensure fast and consistent sample homogenization conditions in online TOC equipment.

## 4. Conclusions

This study confirmed that CULA significantly mitigated the reduction in TOC recovery with increasing SS concentrations and differences in TOC measurement values depending on the TOC oxidation method (UVP, HTC) and sieve size used for sample homogenization. Even as the SS concentration was increased, the TOC recovery of the sample after CULA was secured more than 80% up to 50 mg/L for algae, 30 mg/L for SP, and 200 mg/L for soil. With CULA, the UVP method showed a TOC recovery equivalent to that of the HTC method at an SS concentration range four times larger than that using UL (*p* = 0.17, *n* = 72). The use of CULA was confirmed to improve sample homogeneity by eliminating differences in TOC recovery resulting from the sieve size (100–500 µm, *p* = 0.57, *n* = 72). Based on the correlation between sample homogeneity and turbidity, the turbidity rate (i.e., NTU_5_/NTU_0_) showed a significant relationship (*r*^2^ = 0.73, *p* < 0.01) with the precision (RSD) of the TOC value. Thus, the proposed method can be used to evaluate sample homogeneity and guarantee the reliability of the TOC analysis regardless of the sample origin and pretreatment method, especially in the real-time online application. The CULA pretreatment method can effectively address the major limitations of conventional TOC analysis, such as determining the sample homogeneity, obtaining a high TOC measurement efficiency (TOC recovery and precision), and improving the convenience (SS concentration, oxidation method, sieve size). The results should contribute to the improvement of the reliability of TOC measurements including particulate organic carbons (POCs) in environmental samples. Thus, this study, with a newly proposed TOC analysis procedure including the sample pretreatment by CULA and turbidity-based homogeneity evaluation, should contribute to the field of water quality assessment for particulate pollutants of various origins and the evaluation of the organic carbon cycle and its behavior in aquatic ecosystems.

## Figures and Tables

**Table 1:** Results of the total organic carbon (TOC) and particle size distribution analysis of water samples containing suspended solids (SS) with different origins depending on the pretreatment method. SP—sewage particles, UL—ultrasonic pretreatment, CULA—combined ultrasonic and alkaline pretreatment, RSD—relative standard deviation, DOC—dissolved organic carbon, POC—particulate organic carbon.

Origins	Pretreatment Method	TOC Recovery (%) ^a^	RSD (%) ^b^	DOC/POC ^c^	Particle Size Distribution (%)				
					<100 µm	<200 µm	<300 µm	<500 µm	<1000 µm
Algae	UL ^d^	50.6	21.4	0.96	36.4	57.3	65.0	82.2	96.4
	CULA ^e^	95.4	1.5	11.50	40.1	61.8	70.9	87.3	99.9
SP	UL ^d^	47.6	39.9	0.30	50.6	61.8	67.9	83.8	99.9
	CULA ^e^	90.1	1.9	1.04	96.3	99.9	99.9	99.9	99.9
Soil	UL ^d^	37.4	47.1	0.56	49.5	69.1	76.32	88.08	98.4
	CULA ^e^	77.5	7.9	0.54	87.0	99.9	99.9	99.9	99.9

^a^ Obtained in the pretreatment followed by sieving at 200 µm; ^b^ Relative standard deviation; ^c^ Ratio of DOC to POC in which POC was calculated by TOC–DOC; ^d^ Ultrasonic pretreatment (ultrasonic power: 100%, on/off time: 100 s/5 s, 20 kHz, 10 min); ^e^ Combined ultrasonic and alkaline pretreatment (ultrasonic power: 100%, on/off time: 100 s/5 s, 20 kHz, 0.01 mol/L NaOH, 10 min).

**Table 2:** Summary of two-way analysis of variance (ANOVA) for total organic carbon (TOC) recovery (%) by sieve size and oxidation method for each pretreatment method.

Factor	Condition	*n*	TOC Recovery (%, Mean ± sd) ^c^	*F*-Value	*p*-Value
Ultrasonic pretreatment (UL)					
Sieve size	100 µm	18	35.3 ± 5.7 ^1^	7.57	<0.05
	200 µm	18	42.4 ± 8.5 ^2^		
	300 µm	18	44.8 ± 8.4 ^2^		
	500 µm	18	43.2 ± 8.0 ^2^		
Oxidation method	UVP ^a^	36	36.3 ± 7.2 ^1^	24.81	<0.05
	HTC ^b^	36	45.4 ± 9.3 ^2^		
Sieve size • Oxidation method				0.15	0.85
Combined ultrasonic and alkaline pretreatment (CULA)					
Sieve size	100 µm	18	86.4 ± 8.8 ^1^	0.56	0.57
	200 µm	18	88.4 ± 8.8 ^1^		
	300 µm	18	89.6 ± 8.6 ^1^		
	500 µm	18	87.6 ± 7.6 ^1^		
Oxidation method	UVP ^a^	36	86.4 ± 8.7 ^1^	1.88	0.17
	HTC ^b^	36	89.8 ± 8.7 ^1^		
Sieve size • Oxidation method				0.13	0.87

^a^ UV-persulfate oxidation method; ^b^ High-temperature combustion oxidation method; ^c^ Superscript numbers (^1, 2^) indicate statistical difference (*p* < 0.05).

**Table 3:** Total organic carbon (TOC) recovery (%) and turbidity (NTU) of water samples containing suspended solids with different origins depending on the pretreatment method.

Origins	Method	Time (min)	TOC Recovery (%)				RSD ^d^ (%)	SE ^e^ (%)	NTU_1_/NTU_0_^f^ (%)	NTU_3_/NTU_0_^f^ (%)	NTU_5_/NTU_0_^f^ (%)
			Upper ^a^	Lower ^b^	Mean	ΔTOC ^c^					
Algae	UL ^g^	10	44.29	48.81	46.55	4.52	21.44	17.85	81.3	64.8	50.5
	UL ^g^	30	53.96	54.38	54.17	0.42	10.07	14.22	92.9	89.7	89.7
	CULA^h^	10	95.86	97.79	96.82	1.93	8.28	6.80	86.4	80.3	78.2
	CULA^h^	30	99.46	99.80	99.63	0.34	6.62	6.67	93.0	87.3	85.7
SP	UL ^g^	10	48.65	56.58	52.62	7.93	39.85	8.82	92.0	78.7	61.1
	UL ^g^	30	46.27	48.43	47.35	2.16	12.64	7.79	85.2	78.6	69.6
	CULA ^h^	10	91.32	92.22	91.77	0.90	4.42	2.79	89.3	87.8	87.2
	CULA ^h^	30	93.55	93.80	93.68	0.25	5.58	2.53	92.1	91.5	92.3
Soil	UL ^g^	10	34.56	41.96	38.26	7.40	15.66	13.95	95.7	90.7	82.7
	UL ^g^	30	43.87	45.33	44.60	1.46	13.19	12.51	99.2	96.9	93.5
	CULA ^h^	10	75.20	77.38	76.29	2.18	9.52	12.18	94.3	93.3	88.6
	CULA ^h^	30	76.54	78.36	77.45	1.82	4.52	4.41	96.2	95.6	96.1

^a^ Samples were taken from the upper part of the sample container (i.e., 2/10 position down from the water surface of the container, *n* = 3); ^b^ Samples were taken from the lower part of the sample container (i.e., 2/10 position up from the bottom of the container, *n* = 3); ^c^ Difference in TOC recovery (%) of upper and lower parts of samples in a container; ^d^ Relative standard deviation; ^e^ Standard error of TOC measurement values for samples taken from the upper, middle, and lower parts of the sample container (*n* = 9); ^f^ Percent ratios of turbidity measured after the treated sample was standing for 1, 3, and 5 min (NTU_3_, NTU_3_, NTU_5_) compared to the initial turbidity (NTU_0_); ^g^ Ultrasonic pretreatment; ^h^ Combined ultrasonic and alkali pretreatment.

## References

[1] Byun J.D., Kim T.D., Jung B., Shin T., Kim H. (2010). TOC as a potential index for organic contents of wastewater treatment plant effluents. J. Korean Soc. Environ. Anal..

[2] Costa J.A., Farias N.C., Queirós Y.G.C., Mansur C.R.E. (2013). Determination of oil-in-water using nanoemulsions as solvents and UV visible and total organic carbon detection methods. Talanta.

[3] He W., Chen M., Schlautman M.A., Hur J. (2016). Dynamic exchanges between DOM and POM pools in coastal and inland aquatic ecosystems: A review. Sci. Total Environ..

[4] Yang L., Hur J. (2014). Critical evaluation of spectroscopic indices for organic matter source tracing via end member mixing analysis based on two contrasting sources. Water Res..

[5] Lee H.S., Hur J., Lee M.H., Retelletti B.S., Kim T.W., Shin H.S. (2019). Photochemical release of dissolved organic matter from particulate organic matter: Spectroscopic characteristics and disinfection by-product formation potential. Chemosphere.

[6] Sisouane M., Cascant M.M., Tahiri S., Garrigues S., EL Krati M., EL Kadiri Boutchich G., Cervera M.L., de la Guardia M. (2017). Prediction of organic carbon and total nitrogen contents in organic wastes and their composts by infrared spectroscopy and partial least square regression. Talanta.

[7] Tian Y., Jiang Y., Liu Q., Dong M., Xu D., Liu Y., Xu X. (2019). Using a water quality index to assess the water quality of the upper and middle streams of the Luanhe River, northern China. Sci. Total Environ..

[8] Yoon G.S., Park S.M., Yang H., Tsang D.C.W., Alessi D.S., Baek K.T. (2018). Selection criteria for oxidation method in total organic carbon measurement. Chemosphere.

[9] Zhang J., Yu J., An W., Liu J., Wang Y., Chen Y. (2011). Characterization of disinfection byproduct formation potential in 13 source water in China. J. Environ. Sci..

[10] Bekiari V., Avramidis P. (2013). Data quality in water analysis: Validation of combustion-infrared and combustion-chemiluminescence methods for the simultaneous determination of total organic carbon (TOC) and total nitrogen (TN). Intern. J. Environ. Anal. Chem..

[11] Bisutti I., Hilke I., Schumacher J., Raessler M. (2007). A novel single-run dual temperature combustion (SRDTC) method for the determination of organic, inorganic, and total carbon in soil samples. Talanta.

[12] Dubber D., Gray N.F. (2010). Replacement of chemical oxygen demand (COD) with total organic carbon (TOC) for monitoring wastewater treatment performance to minimize the disposal of toxic analytical waste. J. Environ. Sci. Heal..

[13] Park D.S.M., Lee H.S., Rhee D.S., Shin H.S. (2019). Improvement in the analytical procedure for total organic carbon measurements in particle-containing water samples. J. Korean Soc. Environ. Anal..

[14] Derrien M., Yang L., Hur J. (2017). Lipid biomarkers and spectroscopic indices for identifying organic matter sources in aquatic environments: A review. Water Res..

[15] Osburn C.L., Handsel L.T., Mikan M.P., Paerl H.W., Montgomery M.T. (2012). Fluorescence tracking of dissolved and particulate organic matter quality in a river-dominated estuary. Environ. Sci. Technol..

[16] Aiken G., Kaplan L.A., Weishaar J. (2002). Assessment of relative accuracy in the determination of organic matter concentrations in aquatic systems. J. Environ. Monit..

[17] Kim S.H., Lee H.S., Hur J., Choi B.J., Shin H.S. (2016). Comparison of the efficiency of organic oxidation and the effect of suspended solid particles in the high temperature combustion and UV/persulfate oxidation methods for TOC analysis. J. Korean Soc. Environ. Anal..

[18] ES 04311.1c (2015). Total Organic Carbon-High Temperature Combustion Method.

[19] DIN EN 1484: 1997 (1997). Water Analysis-Guidelines for the Determination of Total Organic Carbon (TOC) and Dissolved Organic Carbon (DOC).

[20] ISO 20236: 2018 (2018). Water Quality-Determination of Total Organic Carbon (TOC), Dissolved Organic Carbon (DOC), Total Bound Nitrogen (TNb) and Dissolved Bound Nitrogen (DNb) after High Temperature Catalytic Oxidative Combustion.

[21] Method 415.2 (1982). Organic Carbon, Total (Low Level) (UV Promoted, Persulfate Oxidation).

[22] Potter B.B., Wimsatt J.C. (2005). Method 415.3-Measurement of Total Organic Carbon, Dissolved Organic Carbon and Specific UV Absorbance at 254 nm in Source Water and Drinking Water.

[23] Jangkorn S., Kuhakaew S., Theantanoo S., Klinla-or H., Sriwiriyarat T. (2011). Evaluation of reusing alum sludge for the coagulation of industrial wastewater containing mixed anionic surfactants. J. Environ. Sci..

[24] Jin Y., Li H., Mahar R.B., Wang Z., Nie Y. (2009). Combined alkaline and ultrasonic pretreatment of sludge before aerobic digestion. J. Environ. Sci..

[25] Tian X., Trzcinski A.P., Lin L., Ng W.J. (2016). Enhancing sewage sludge anaerobic “re-digestion” with combinations of ultrasonic, ozone and alkaline treatments. J. Environ. Chem. Eng..

[26] Wang H., Wang Y., Zhuang W.-E., Chen W., Shi W., Zhu Z., Yang L. (2020). Effects of fish culture on particulate organic matter in a reservoir-type river as revealed by absorption spectroscopy and fluorescence EEM-PARAFAC. Chemosphere.

[27] He W., Jung H., Lee J.-H., Hur J. (2016). Differences in spectroscopic characteristics between dissolved and particulate organic matters in sediments: Insight into distribution behavior of sediment organic matter. Sci. Total Environ..

[28] Lu D., Xiao K., Chen Y., Soh Y.N.A., Zhou Y. (2018). Transformation of dissolved organic matters produced from alkaline-ultrasonic sludge pretreatment in anaerobic digestion: From macro to micro. Water Res..

[29] Lee H.S., Hur J., Shin H.S. (2020). Enhancing the total organic carbon measurement efficiency for water samples containing suspended solids using alkaline and ultrasonic pretreatment methods. J. Environ. Sci..

[30] Kim S.H., Park H., Chung H.M., Jeong D.H., Hur J., Shin H.S. (2016). A study on the pretreatment for TOC determination in suspended solid-containing samples. J. Korean Soc. Environ. Anal..

[31] Lu X., Zhou F., Chen F., Lao Q., Zhu Q., Meng Y., Chen C. (2020). Spatial and seasonal variations of sedimentary organic matter in a subtropical bay: Implication for human interventions. Int. J. Environ. Res. Public Health.

